# Internet search patterns reveal firearm sales, policies, and deaths

**DOI:** 10.1038/s41746-020-00356-6

**Published:** 2020-11-20

**Authors:** John S. Brownstein, Adam D. Nahari, Ben Y. Reis

**Affiliations:** 1 Computational Epidemiology Lab, Boston Children’s Hospital, Boston, MA USA; 2 Harvard Medical School, Boston, MA USA; 3 Predictive Medicine Group, Computational Health Informatics Program, Boston Children’s Hospital, Boston, MA USA; 4 Harvard University, Cambridge, MA USA

**Keywords:** Epidemiology, Translational research

## Abstract

Firearm-related violence is a leading cause of morbidity and mortality and is at the center of a major public health and policy debate in the United States. Despite the critical role of data in informing this debate, accurate and comprehensive data on firearm sales and ownership is not readily available. In this study, we evaluate the potential of using firearm-related internet search queries as a complementary, freely available, and near-real-time data source for tracking firearm sales and ownership that enables analysis at finer geographic and temporal scales. (Here, we examine data by state and by month to compare with other data sources, but search engine volume can be analyzed by city and by the week or by day). We validate search query volume against available data on background checks in all 50 US states, and find that they are highly correlated over time (Pearson’s *r* = 0.96, Spearman’s *ρ* = 0.94) and space (Pearson’s *r* = 0.78, Spearman’s *ρ* = 0.76). We find that stratifying this analysis by gun type (long-gun vs. handgun) increases this correlation dramatically, across both time and space. We also find a positive association between firearm-related search query volume and firearm-related mortality (Pearson’s *r* = 0.87, Spearman’s *ρ* = 0.90), and a negative association with the strength of state-level firearm control policies (Pearson’s *r* = −0.82, Spearman’s *ρ* = −0.83). Based on these findings, we propose a framework for prospective surveillance that incorporates firearm-related internet search volume as a useful complementary data source to inform the public health policy debate on this issue.

## Introduction

Firearm-related violence is a major source of morbidity and mortality in the United States, with an average of 36,383 deaths and 100,120 injuries per year between 2013 and 2017^1^. High profile mass shootings further fuel the ongoing debate on firearm policy. Central to this debate is the challenge of understanding the impact of policy on firearm ownership and firearm-related morbidity and mortality. Despite the critical role of data in understanding these policy questions, accurate and comprehensive data on gun sales and ownership are not available. Current research on firearms typically relies on data derived from surveys, proxy variables, or production and import data.

The most common data source used in firearm policy research is the Federal Bureau of Investigation’s National Instant Criminal Background Check System (NICS)^2,3^. While this information is a useful surrogate measure, it does not represent actual firearm sales and is greatly impacted by regulations that vary from state to state. In some cases, NICS data may represent an overestimation due to permit denials, multiple background checks conducted for single firearm purchases, or waiting periods that deter eventual firearm sales. In other cases, NICS data may underestimate firearm sales due to multiple firearm purchases for a single background check, exemptions from background checks based on concealed handgun permits, and lack of information on sales by private sellers, including those conducted at gun shows. Federal provisions that limit certain agencies from engaging in gun control research and tracking have further hindered accurate firearm surveillance^4,5^.

The central importance of the gun control debate in the public sphere, together with the current restrictions on data-gathering, drive the need for alternative, low-cost and timely sources of firearm-related data. In recent years, a new generation of public health surveillance efforts has relied on patterns of online searches^6^. Search data can be used to rapidly examine population health and evaluate the impact of health policies. Some recent examples of the use of search data for surveillance purposes include influenza (Pearson’s *r* = 0.91)^7^, dengue fever (Pearson’s *r* of between 0.82 and 0.99)^8^, abortion (Spearman’s *ρ* of between −0.48 and −0.55)^9^, smoking (large increases in search volume reported, no correlations calculated)^10^ and mental health (wavelet phase analysis used to isolate seasonal components, seasonal percentage changes in search volume reported)^11^.

Studies analyzing gun-related Internet searches have found that U.S. search volume for the term “buy gun” is correlated to the number of firearm background checks performed in the U.S. between 2008 and 2015 (Pearson’s *r* = 0.84)^12^. Studies have also found that search volume for firearm-related terms changes in response to mass shootings^5,12–18^. These studies measured overall increases in the volume of search terms before and after mass shootings and found increases of tens or even hundreds of percent. One recent study found an increase in gun-related searches during the COVID-19 pandemic^16^.

The present study extends the work of these previous studies by conducting an in-depth analysis of gun-related search volumes and their relationships to different gun-related phenomena. We begin by comparing search query volume for a range of gun-related search terms against available background-check data over both time and space. We also stratify these analyses by gun type. We then examine the relationship of these data to state-level firearm-related mortality and state-level firearm policy. To our knowledge, this is the first study to explore the relationship between firearm-related search volume and firearm-related mortality, as well as the restrictiveness of local firearm policies. Finally, we propose a framework for prospective surveillance that incorporates gun-related internet search volume as a freely available, real-time complementary data source that enables analysis at finer geographic and temporal scales, with fewer delays in data collection, to inform the public policy debate.

## Results

### Firearm background checks and search data are correlated

**Fig. 1:**
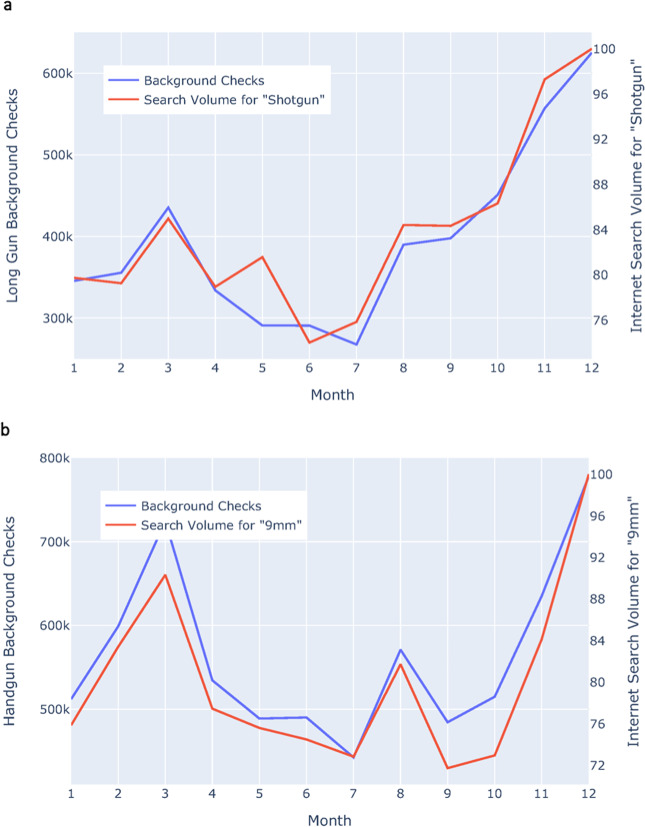
Monthly comparison of background checks vs. search volumes. **a** U.S. long-gun background checks vs. U.S. relative Internet search volume for the term *shotgun* (Pearson’s *r* = 0.96, Spearman’s *ρ* = 0.94) and **b** U.S. handgun background checks vs. U.S. relative Internet search volume for the term 9 mm in 2019 (Pearson’s *r* = 0.97, Spearman’s *ρ* = 0.94).

The correlation between search volume for the general term *gun* and the total number of background checks in the U.S. yielded a Pearson’s *r* of 0.74 and Spearman’s *ρ* of 0.62 (*P* = 0.006). We investigated improving this correlation by stratifying the analysis by gun type—i.e., long-guns vs. handguns. The results improved significantly. Figure 1a shows the relationship over time between the number of U.S. long-gun background-checks and U.S. internet search volume for the term *shotgun* in 2019 (Pearson’s *r* = 0.96, Spearman’s *ρ* = 0.94, *P* < 0.001). Figure 1b shows the relationship over time between the number of U.S. handgun background-checks and U.S. internet search volume for the term 9 mm in 2019 (Pearson’s *r* = 0.97, Spearman’s *ρ* = 0.94, *P* < 0.001).

**Fig. 2:**
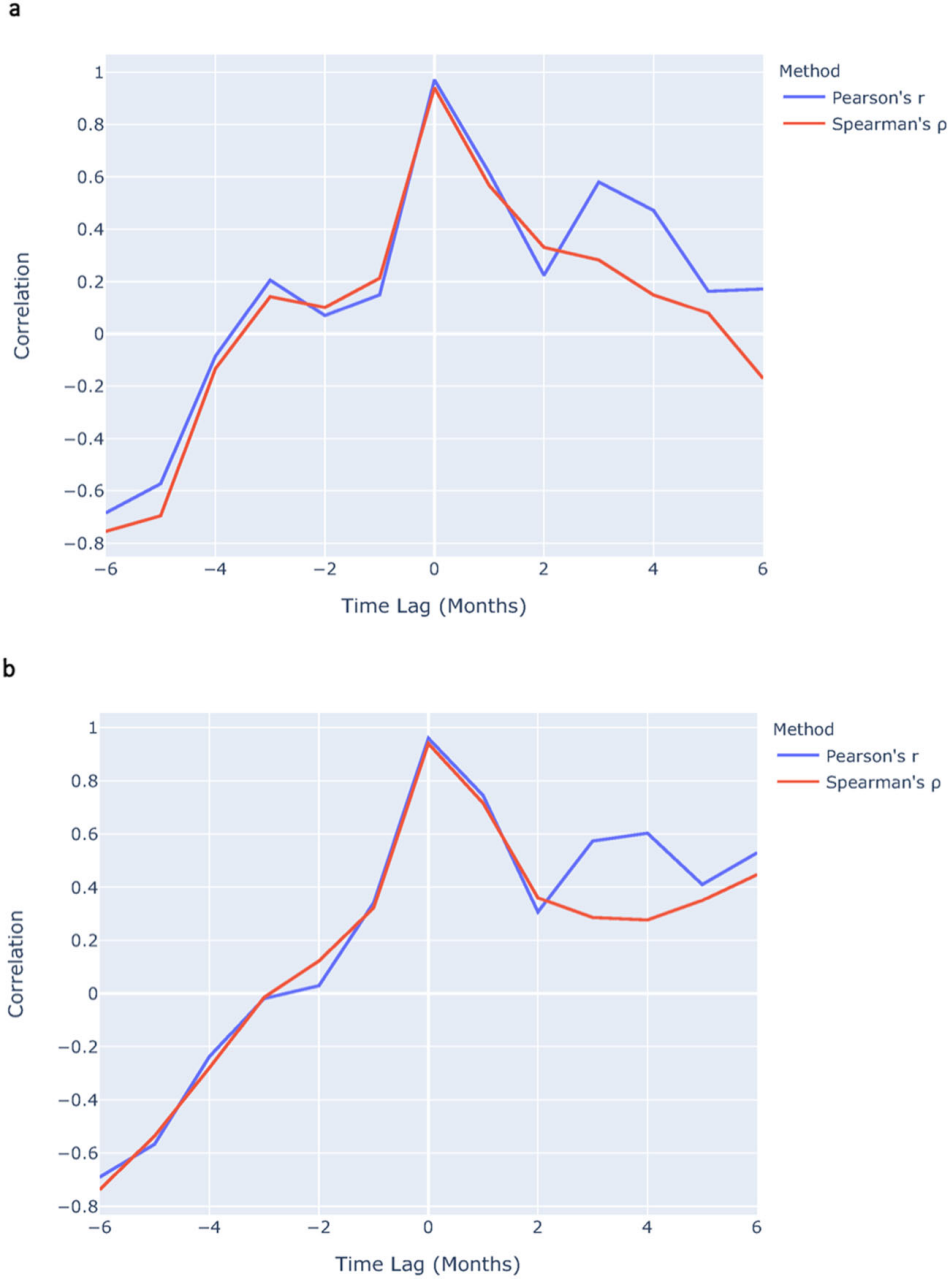
Time-lagged correlations of search volume vs. backgrround checks. **a** Internet search volume for “shotgun” and long-gun background checks in 2019. **b** Internet search volume for “9 mm” and handgun background checks in 2019.

Figure 2 shows time-lagged correlation plots of internet search volume for “shotgun” and long-gun background checks in 2019, as well as internet search volume for “9 mm” and handgun background checks in 2019. Examining a range of lags between −6 months and +6 months, the time-lagged correlation analysis reveals that the highest correlations were achieved at a zero-time lag.

**Fig. 3:**
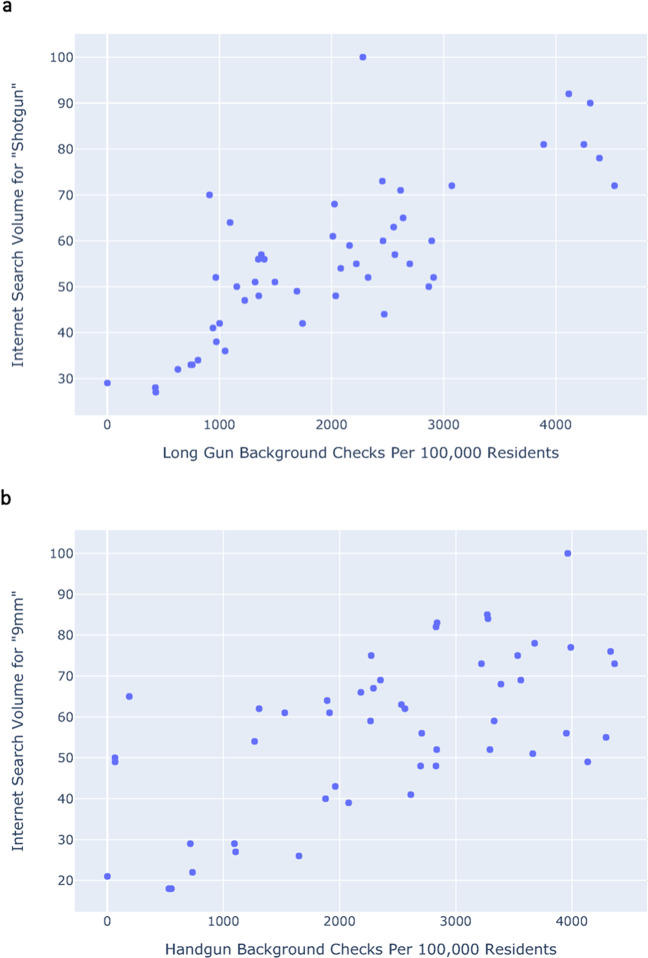
State-by-state comparisons of background checks vs. search volumes. **a** U.S. long-gun background checks vs. U.S. relative Internet search volume for the term *shotgun* (Pearson’s *r* = 0.78, Spearman’s *ρ* = 0.76). **b** U.S. handgun background checks vs. U.S. relative search Internet search volume for the term 9 mm (Pearson’s *r* = 0.63, Spearman’s *ρ* = 0.59) across all 50 US states in 2019.

Next, we examined this association over space: The correlation between the total number of background-checks per 100,000 residents and internet search volume for the general term *gun* across 50 US states during 2019 yielded a Pearson’s *r* of 0.34 and a Spearman’s *ρ* of 0.66 (*P* = 0.017). Stratification by gun type improved this correlation significantly: Fig. 3a shows the relationship between the number of long-gun background checks per 100,000 residents and internet search volume for the term *shotgun* across 50 US states during 2019 (Pearson’s *r* = 0.78, Spearman’s *ρ* = 0.76, *P* < 0.001), and Fig. 3b shows the relationship between the number of handgun background-checks per 100,000 residents and Internet search volume for the term 9 mm across 50 US states during 2019 (Pearson’s *r* = 0.63, Spearman’s *ρ* = 0.59, *P* < 0.001).

### Firearm-related deaths and search data are correlated

**Fig. 4:**
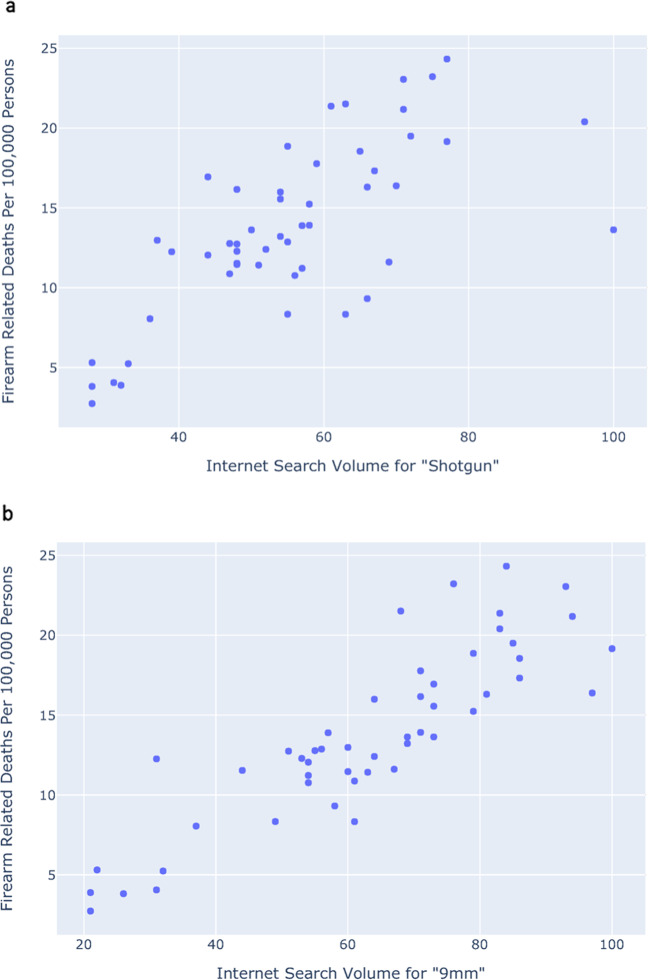
State-by-state comparisons of firearm deaths vs. search volumes. **a** Firearm-related deaths per 100,000 people vs. relative Internet search volume for *shotgun* (Pearson’s *r* = 0.71, Spearman’s *ρ* = 0.68). **b** Firearm-related deaths per 100,000 people vs. relative Internet search volume for 9 mm (Pearson’s *r* = 0.87, Spearman’s *ρ* = 0.90) across all 50 US states in 2017.

Next, we analyzed the association between firearm-related deaths and search data across time and space: Fig. 4a shows the relationship between rates of firearm-related deaths and internet search volume for the term *shotgun* across all 50 US states in 2017, the year with the most recently available data on firearm-related deaths (Pearson’s *r* = 0.71, Spearman’s *ρ* = 0.68, *P* < 0.001). Other search terms yield even stronger correlations, including 9 mm (Pearson’s *r* = 0.87, Spearman’s *ρ* = 0.90, *P* < 0.001; Fig. 4b).

**Fig. 5:**
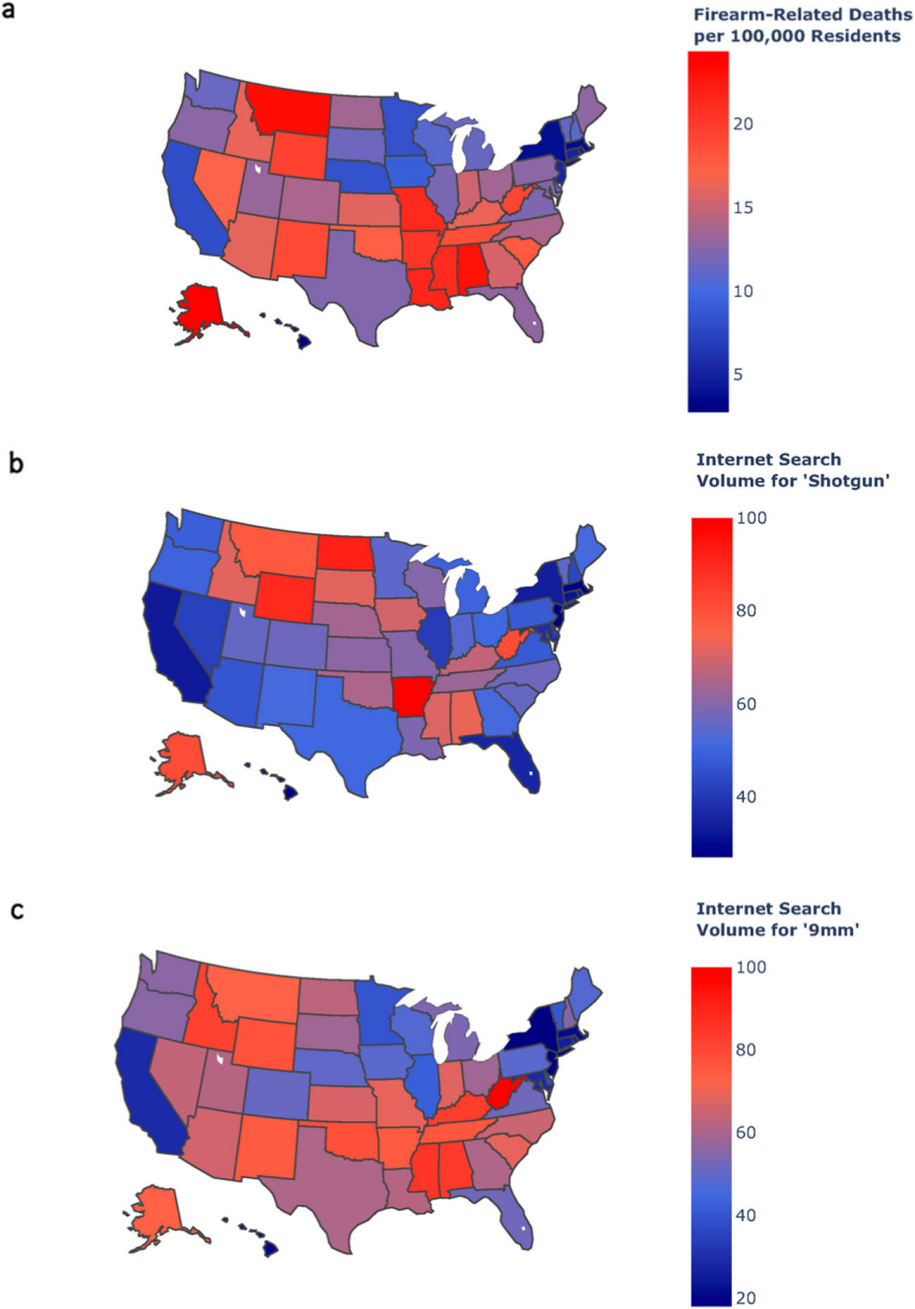
Geographic view of firearm deaths and search volumes. **a** Firearm-related deaths per 100,000 residents **b** Relative search volume for the term *shotgun*. **c** Relative search volume for the term 9mm across all 50 US states in 2017. All maps produced with Plotly version 4.11, available through an MIT License (https://github.com/plotly/plotly.js/blob/master/LICENSE).

Figure 5a shows a geographical representation of firearm-related deaths per 100,000 residents across all 50 US states in 2017. Figure 5b shows a similar geographical representation of internet search volume for the term *shotgun* across all 50 US states in 2017, and Fig. 5c shows the geographical representation of internet search volume for the term 9 mm across all 50 US states in 2017.

### The restrictiveness of state gun laws and search data are inversely correlated

**Fig. 6:**
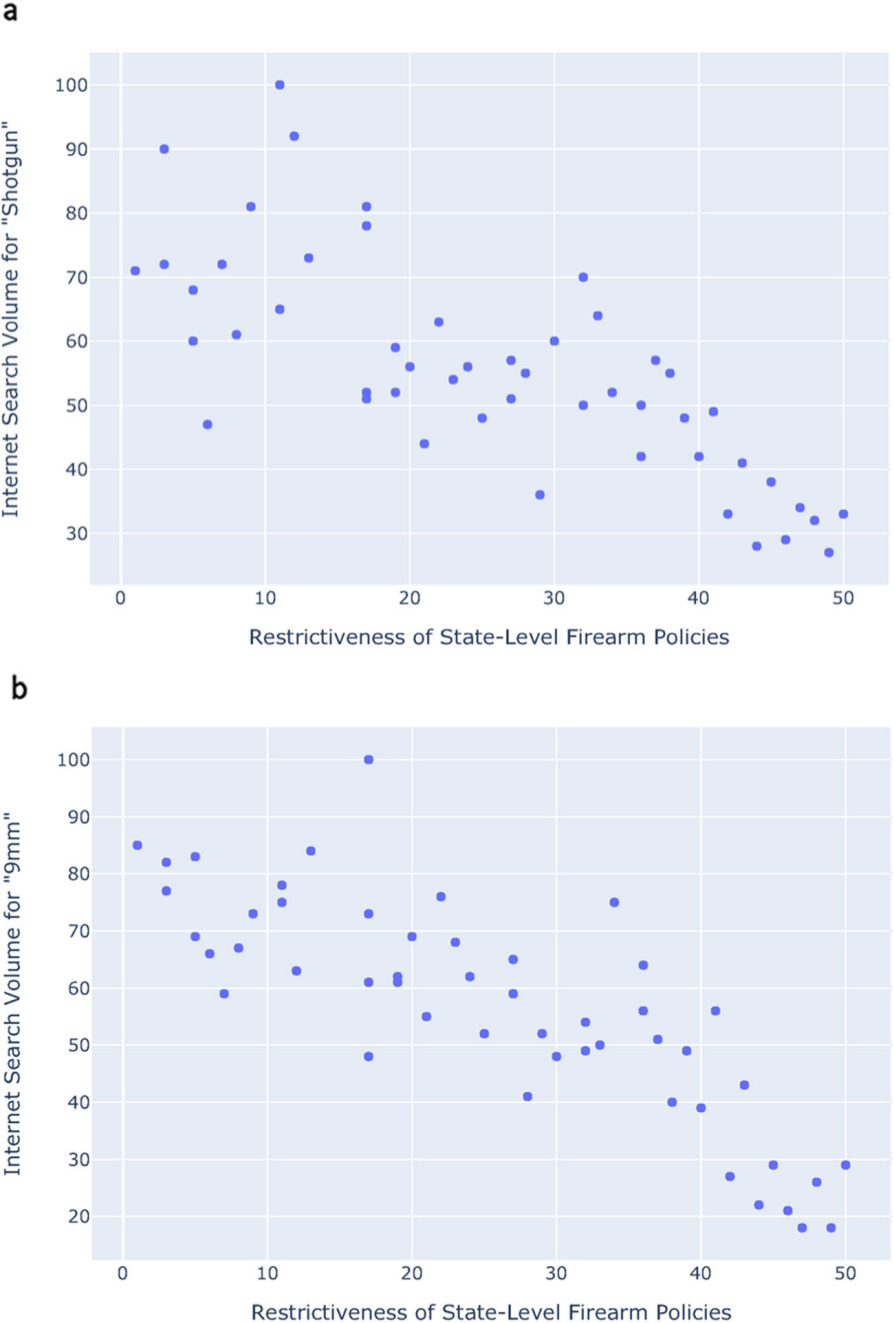
State-level firearm policies vs. search volume. **a** Restrictiveness of state-level firearm policies, based on The Giffords Law Center to Prevent Gun Violence 2019 Annual Gun Law State Scorecard (1–50 scale) vs. Internet search volume for the terms *shotgun* (Pearson’s *r* = 0.76, Spearman’s *ρ* = −0.78). **b** The same analysis shown for the search term 9 mm (Pearson’s *r* = −0.82, Spearman’s *ρ* = −0.83). All data from 2019.

**Fig. 7:**
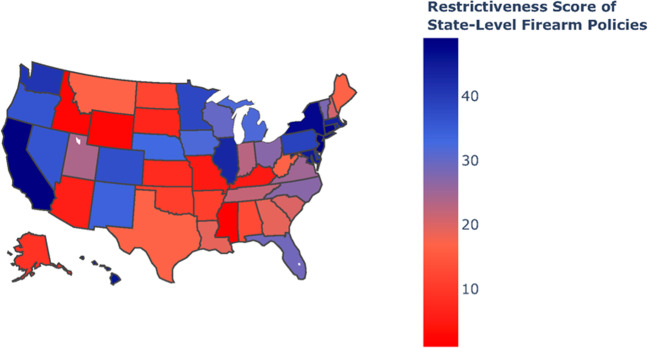
Geographic view of state-level firearm policies. Restrictiveness of state-level firearm policies across all 50 US states based on The Giffords Law Center to Prevent Gun Violence 2019 Annual Gun Law State Scorecard (1–50 scale). All maps produced with Plotly version 4.11, available through an MIT License (https://github.com/plotly/plotly.js/blob/master/LICENSE).

We analyzed the association between state gun laws and firearm-related search queries. Figure 6a shows the relationship between the restrictiveness of state-level firearm policies and internet search volume for the term *shotgun* across all 50 US states in 2019 (Pearson’s *r* = 0.76, Spearman’s *ρ* = −0.78, *P* < 0.001). Other search terms were also found to be strongly negatively correlated, including 9 mm (Pearson’s *r* = −0.82, Spearman’s *ρ* = −0.83, *P* < 0.001; Fig. 6b). Figure 7 shows a geographical representation of the restrictiveness of state-level firearm policies across all 50 US states, based on The Giffords Law Center to Prevent Gun Violence 2019 Annual Gun Law State Scorecard, rated on a 1–50 scale.

**Fig. 8:**
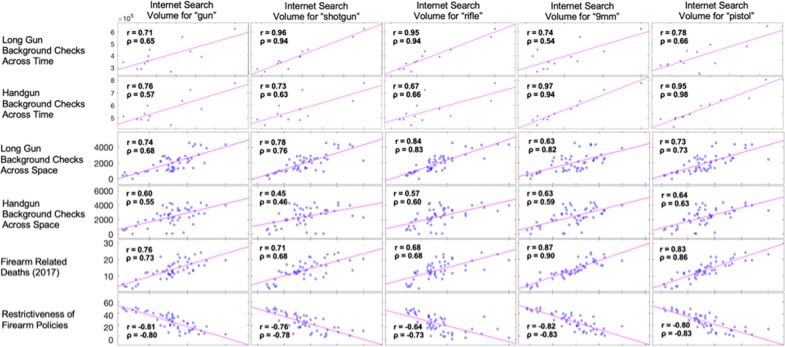
Analysis and search term correlation plots. Correlation plots and corresponding Pearson’s r and Spearman’s *ρ* values for all analyses and all search terms.

While the above figures highlight specific search terms with the highest correlations for each analysis, Fig. 8 shows a summary correlation plot with Pearson’s *r* and Spearman’s *ρ* values for all correlation analyses across all search terms.

## Discussion

The results of this study indicate that firearm-related Internet search query volume is strongly correlated to background checks over both time and space and that they are most strongly temporally correlated at zero months lag. Since background checks are currently the standard proxy used for tracking firearm sales, the strong correlation with background checks suggests that search queries could be used as a complementary data source for tracking firearm sales. We found that the correlation is especially strong when stratifying by gun type: examining the number of long-gun background checks against long-gun-related search queries such as *shotgun* and *rifle*, and the number of handgun background checks with handgun related search queries such as *pistol* and 9 mm.

To the best of the authors’ knowledge, this is the first study to analyze the relationships between firearm-related search terms and firearm-related deaths and state-level firearm policy. We find that an increased interest in firearms is associated not only with higher firearm-related mortality rates but also with less restrictive firearm policies. These findings suggest that firearm-related Internet search data can be used to identify salient trends and correlations related to both public health and policy issues. Further studies could explore how more granular firearm policy components are associated with firearm-related search volume.

Information on Internet search volume is freely available, appears in near-real-time, and has greater temporal and spatial resolution than other available sources. Furthermore, search data may capture information that is not captured by more formal or official data collection efforts. For example, background check data do not typically include information on sales by private sellers, including those conducted at gun shows, while Internet search data do not have this limitation. In this way, changes or relative differences in firearm-related Internet search volume could be used to track the level of interest, the usage, or the sale of firearms across time and space. Firearm-related Internet search volume could also be used to monitor the effects of certain policy decisions or public health efforts, such as changes to local gun regulations, or gun safety educational initiatives.

Due to current regulatory limitations on the collection of firearm data, no gold standard exists for measuring firearm sales or ownership. We, therefore, compared search volume on firearm-related terms to the widely used proxy of firearm background checks. While the application of search query data to understanding public health policy remains a promising avenue of exploration, there are also certain limitations to this approach. Despite covering a large segment of the population, these data do not offer universal coverage, as access to the Internet varies by demographic, socioeconomic, and geographic factors. Furthermore, the specific intent motivating each user’s search is not known. For example, the search for a *shotgun* may be driven by a multitude of reasons other than purchasing a shotgun, such as media coverage of gun-related violence following mass shootings. Tracking multiple search terms can aid in increasing the specificity of the signal. Levine and McKnight, for example, used search volume for the terms “*buy gun”* and “*clean gun”* to better understand the motivating factors behind searches^12^. Further validation studies would be beneficial, as the data represent a unique opportunity to gain broad population-scale insight in real-time.

The results of this study suggest that Internet search patterns can be a valuable, timely, and complementary resource for tracking firearm sales and ownership that can be put to practical use today to inform an important public health and policy debate.

## Methods

### Data retrieval

In order to calculate background-check rates per 100,000 residents for each U.S. state in 2019, we retrieved data from the Federal Bureau of Investigation’s NICS^19^ on background-checks initiated by an officially-licensed Federal Firearms Licensee or criminal justice/law enforcement agency prior to the issuance of a long gun or handgun permit. We retrieved state population estimates from the US census^20^. We also retrieved state-level data on firearm-related deaths during 2017 from the Centers for Disease Control and Prevention^1^, and data on the restrictiveness of state-level firearm policies from The Giffords Law Center to Prevent Gun Violence (https://lawcenter.giffords.org/scorecard). Finally, we retrieved data on internet search volume from Google Trends (https://trends.google.com/trends/?geo=US).

In order to determine whether there were search terms correlated to gun violence, we examined a range of common search terms related to guns and performed correlation analyses for each of them. We highlight specific terms with the greatest correlations in the figures below and present the results for all search terms in Fig. 8. All data were retrieved on February 10, 2020, and the most recently available data was used for each analysis performed.

### Data analysis

We conducted correlation analyses between different search terms and a range of gun-related variables, including background checks (stratified by type of gun—long guns vs. handguns) over both time and space, gun-related deaths for all 50 states, and restrictiveness of gun policies for all 50 states. We also conducted a time-lagged correlation analysis where we analyzed the effects of shifting the search data by between −6 and +6 months relative to the background check data.

For the correlation analyses, we used both Pearson’s *r* and Spearman’s *ρ*. Each of these measures of association has its own strengths and weaknesses. The parametric Pearson’s *r* utilizes more information than the nonparametric Spearman’s *ρ* (by considering the actual size of the changes in both variables rather than simply their rank), but Pearson’s *r* is also more vulnerable to outliers. We, therefore, report both measures of association here in order to provide a more comprehensive picture of the association between variables.

### Reporting summary

Further information on research design is available in the Nature Research Reporting Summary linked to this article.

## Supplementary information

Reporting Summary

## Data Availability

The gun sales data that support the findings of this study are available from the Federal Bureau of Investigation’s National Instant Criminal Background Check System (NICS), https://www.fbi.gov/file-repository/nics_firearm_checks_-_month_year_by_state_type.pdf/view. The gun search volume data that support the findings of this study are available from Google Trends, https://trends.google.com/trends/?geo=US. The reported firearm fatality data that support the findings of this study are available from The Centers for Disease Control and Prevention’s Web-based Injury Statistics Query and Reporting System, https://www.cdc.gov/injury/wisqars. The state population data that support the findings of this study are available from the United States Census Bureau’s State Population Totals and Components of Change: 2010–2019, https://www.census.gov/data/tables/time-series/demo/popest/2010s-state-total.html.

## Code availability

All analyses were performed using the publicly available Python programming language, version 3.8, with the Python packages Pandas version 1.1.3 and Plotly version 4.11. All maps produced with Plotly version 4.11, available through an MIT License (https://github.com/plotly/plotly.js/blob/master/LICENSE).
